# Combination of pharmacophore hypothesis, genetic function approximation model, and molecular docking to identify novel inhibitors of S6K1

**DOI:** 10.1007/s11030-013-9473-7

**Published:** 2013-08-28

**Authors:** Hui Zhang, Ming-Li Xiang, Jun-Yu Liang, Tao Zeng, Xiao-Nuo Zhang, Ji Zhang, Sheng-Yong Yang

**Affiliations:** 1 College of Life Science, Northwest Normal University, Lanzhou , 730070 Gansu People’s Republic of China; 2 State Key Laboratory of Biotherapy and Cancer Center, West China Hospital, West China Medical School, Sichuan University, Chengdu , 610041 Sichuan People’s Republic of China

**Keywords:** S6K1, Pharmacophore model, Genetic function approximation model, Molecular docking, Virtual screening

## Abstract

S6K1 has emerged as a potential target for the treatment for obesity, type II diabetes and cancer diseases. Discovery of S6K1 inhibitors has thus attracted much attention in recent years. In this investigation, a hybrid virtual screening method that involves pharmacophore hypothesis, genetic function approximation (GFA) model, and molecular docking technology has been used to discover S6K1 inhibitors especially with novel scaffolds. The common feature pharmacophore hypothesis and GFA regression model of S6K1 inhibitors were first developed and applied in a virtual screen of the Specs database for retrieving S6K1 inhibitors. Then, the molecular docking method was carried out to re-filter these screened compounds. Finally, 60 compounds with promising S6K1 inhibitory activity were carefully selected and have been handed over to the other group to complete the follow-up compound synthesis (or purchase) and activity test.

**Electronic supplementary material:**

The online version of this article (doi:10.1007/s11030-013-9473-7) contains supplementary material, which is available to authorized users.

## Introduction

The 70-kDa ribosomal S6 kinase 1 (S6K1) is an important downstream effectors of mTOR distributed in the nucleus and cytoplasm [1, 2], which mediates a variety of cellular processes, including protein synthesis, mRNA process, glucose homeostasis, cell growth and survival [3–5]. Presently, S6K1 was found to be over-expressed in various tumors, including breast cancer and brain tumor [6–8]. In addition, recent studies have demonstrated that insulin and nutrients could activate S6K1, while the prolonged activation of S6K1 could hyperphosphorylate IRS1 (insulin receptor substrate 1) via a negative feedback loop way, disrupting interactions between IRS1 and IR (insulin receptor) and leading to the development of insulin resistance [9–13]. Moreover, studies in mammalian models have approved that inhibition of S6K1 function could reduce adiposity and the risk of age-related pathologies, increase insulin sensitivity, and prolong mammalian life span [14–16]. Thus, developing S6K1-specific inhibitors could offer an effective tool for the treatment of obesity, type II diabetes and cancers.

Over the past few years, many academic institutions and pharmaceutical companies have realized the importance of S6K1 in human diseases and attempted to develop S6K1-specific inhibitors, and more than seventy inhibitors have been reported to date [16–18]. Unfortunately, among these reported inhibitors, only the oltipraz has entered Phase II clinical trials for inhibiting the development of insulin resistance, hyperglycemia and fatty acid synthesis [19], and the others have just moderate or weak inhibition potency. Therefore, pursuing and discovering more potent and selective S6K1 inhibitors with new scaffolds is very necessary.

High-throughput screening methods, especially the computer-aided virtual screening (VS), could provide economical and rapid approaches for the discovery of lead compounds with new scaffolds of specified targets from large chemical databases. Docking-based VS (DB-VS) and pharmacophore-based VS (PB-VS) classical methods have been widely used to find hit compounds in drug discovery [20, 21]. However, previous studies have shown that the individual use of these methods generally lead to low hit rate and low enrichment factor, as well as high false positive rate. On the other hand, the combined use of VS methods in a hybrid protocol would overcome these drawbacks [22–26].

Thus, in this work, we developed a combined VS method, including HipHop method, GFA model [27, 28] and molecular docking method, to identify S6K1 novel inhibitors. The GFA algorithm is derived from Rogers’ G/SPLINES algorithm that combined Friedman’s multivariate adaptive regression splines algorithm and Holland’s genetic algorithm to evolve a population of equations, which has been widely applied to the prediction of biological activity by using physicochemical properties of a series of compounds [26–30]. In this investigation, we shall first develop a common feature pharmacophore hypothesis and GFA-based VS (GB-VS) model. Then, PB-VS, GB-VS and DB-VS would be sequentially applied to screen the Specs database. Finally, potential active compounds were selected and handed over to other research group to complete the follow-up compound synthesis (or purchase) and activity test.

## Methods and materials

### Common chemical feature-based pharmacophore modeling

**Fig. 1:**
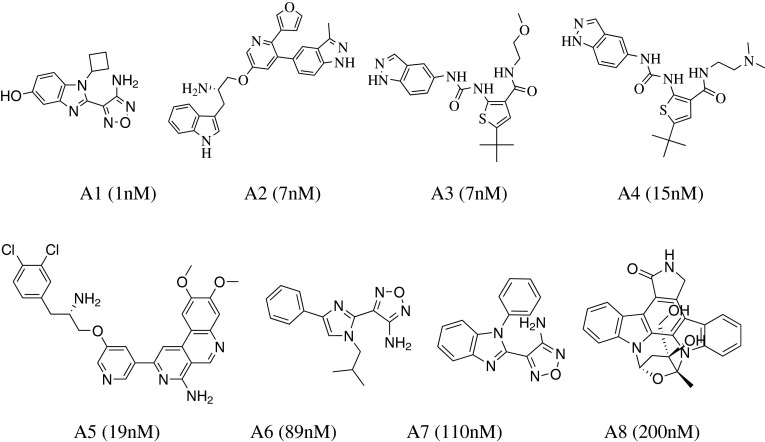
Chemical structures of S6K1 kinase inhibitors in the training set together with their biological activity data (IC50) for HipHop run

The HipHop algorithm implemented in the Accelrys Discovery Studio 3.1 program package (Accelrys Inc., San Diego, CA) was employed for pharmacophore modeling. Eight S6K1 inhibitors (A1–A8), based on a wide range of biological activities and structural diversity, were chosen to form the training set (Fig. 1). Compound A1 was selected as “reference compound”, and their “principal” and “MaxOmitFeat” values were assigned as 2 and 0, respectively. The other compounds (A2–A8) of the training set were set to 1 for “Principal” and “MaxOmitFeat” values. The “minimum Interfeature Distance” value was set to 2.97 Å. Five features (hydrogen bond acceptor, hydrogen bond donor, hydrophobic, hydrophobic-aliphatic and ring-aromatic) were initially selected and used for pharmacophore generation. The parameters “Min” and “Max” of the hydrophobic feature were defined as 1 and 5, respectively. All the other parameters were kept at their default values.

### GFA model

The GFA model was developed using Discovery Studio (DS) version 3.1 (Accelrys Inc., San Diego, CA). All collected 73 inhibitors were randomly divided into a training set and a test set. The training set consisted of 55 agents, and the remaining 18 agents were used as a test set. The “Model Form” was chosen as Full Quadratic, “Maximum Equation Length” was set to 27. The parameters for Population Size, Maximum Generations, Scoring Method and Scoring Function were set to 1,000, 50,000, Pareto (NSGA-II) and R-squared, respectively. “Model Domain Fingerprint” was defined as ECFP_6. “Maximum Correlation” was set to 0.95. The other parameters were kept at their default values.

### Molecular docking study

All the molecular docking studies were carried out by genetic optimization of ligand docking that adopts the genetic algorithm to dock flexible ligands into protein binding sites. The crystal structure (PDB: 3A60) of the kinase domain bound to the inhibitor staurosporine was employed in the docking studies. All water molecules were deleted, and all hydrogen atoms were added to the protein by using Accelrys Discovery Studio 3.1. The binding site was defined as a sphere containing the residues within 7.8 Å from the ligand, which is large enough to cover the ATP binding region at the active site.

### Cluster ligands model

The cluster ligands model was run in the Accelrys Discovery Studio (DS) version 3.1. The “Number of Clusters” and “predefined set” were defined as 10 and FCFP-6, respectively.

## Results and discussion

### Generation of common pharmacophore models of S6K1 inhibitors

**Fig. 2:**
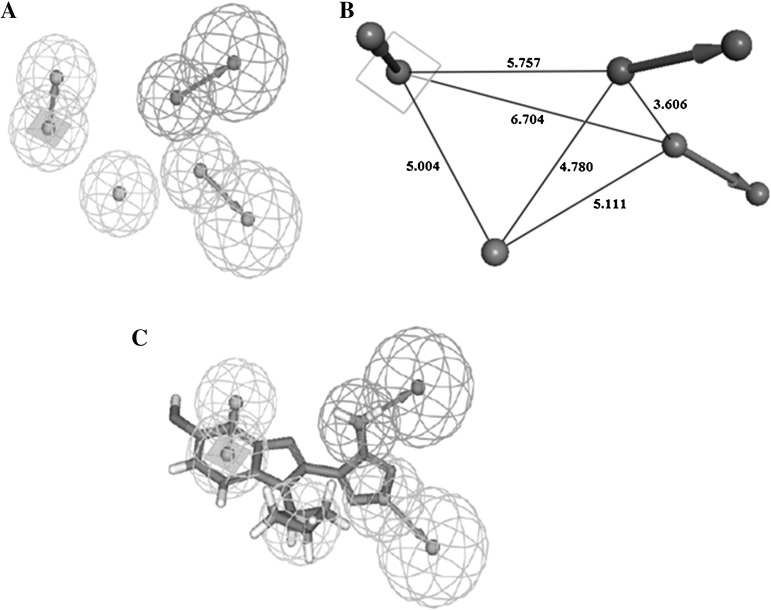
**a** Best pharmacophore model of S6K1 inhibitors generated by HipHop. **b** 3D spatial relationship and geometric parameters of Hypo1. **c** The best HipHop model aligned with one of the most active compounds 1 (IC50 $=$ 1 nM) in the training set. The features are color coded: *orange* ring-aromatic, *green* hydrogen-bond acceptor, *magenta* hydrogen-bond donor, *cyan* hydrophobic feature. (Color figure online)

Eight inhibitors, containing different scaffolds and activities, were used to generate common pharmacophore features. Finally, a total of ten pharmacophore models were generated by using HipHop algorithm. Figure 2a shows the best pharmacophore model, Hypo1, with four features: one hydrogen bond acceptor, one hydrogen bond donor, one hydrophobic feature, and one ring-aromatic feature. The 3D space and distance constraints of these pharmacophore features are presented in Fig. 2b. The hydrophobic feature is far from the centers of ring-aromatic feature, hydrogen bond donor and hydrogen bond acceptor by 5.004, 4.780, and 5.111 Å, respectively. The hydrogen bond donor feature is far from the ring-aromatic feature and hydrogen bond acceptor by 5.757 Å and 3.606 Å. And the centers of the ring-aromatic feature and hydrogen bond acceptor feature are separated by 6.704 Å. Figure 2c shows the most active inhibitor mapped with the pharmacophore features. Clearly, inhibitor A1 is mapped very well (fit value: 3.99) with these features of Hypo1. Furthermore, in order to further validate the established pharmacophore model, all the collected inhibitors (73 compounds) were mapped on Hypo1. The results showed 84.9 % inhibitors were mapped with the features of Hypo1 (fit value >2.5). Taken together, this demonstrates the established pharmacophore model is in line with the features of S6K1 inhibitors.

### Development and validation of the GFA regression model

**Fig. 3:**
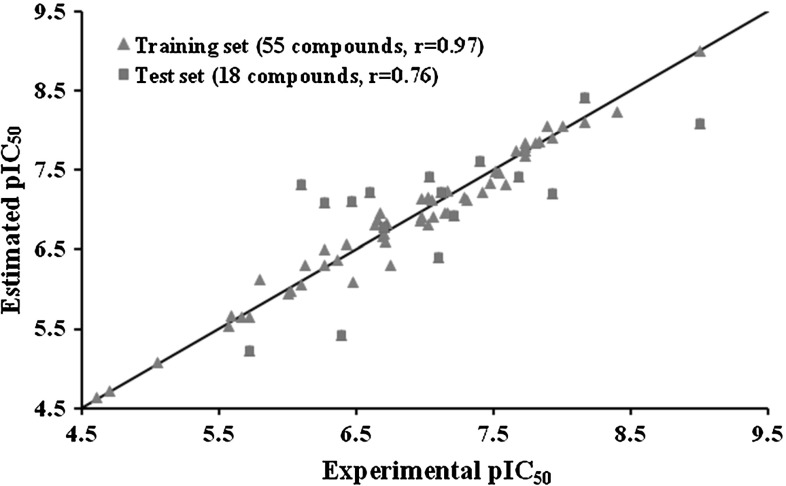
Plot of the correlation between the experimental activity and the estimated activity by the best GFA model for the training set and test set compounds

Fifty five compounds were used to train the GFA models and the remaining 18 compounds were used as a test set to evaluate the capacity of GFA models. Eight molecular property descriptors (ALogP, Molecular_Weight, Num_H_Donors, Num_H_Acceptors, Num_RotatableBonds, Num_Rings, Num_AromaticRings and Molecular_FractionalPolarSurfaceArea) and one structural fingerprint descriptor (ECFP_6) were employed in building the GFA models. Finally, ten GFA models were generated. The following criteria were used to evaluate the produced models capacity and suitability: (a) the lack of fit (LOF) score, (b) variable terms in the equation, and (c) the internal and external predictive ability of the equation. One GFA model showed greater correlation coefficient, lowest LOF and least possible intervariable correlation comparatively was selected to predict activity, in which five descriptors were finally selected to construct the GFA model equation (Molecular_Weight, Number_H_Donors, Alogp, Molecular_FractionalPolarSurfaceArea and ECFP_6). The correlation coefficients of the training set and test set are 0.97 and 0.76, respectively. Figure 3 shows the experimental VS estimated pIC50 of the training set and test set molecules for S6K1.

### Parameter setting and scoring function selection for the docking study

In molecular docking, parameters and scoring functions seriously influence the accuracy of VS. Thus, we carried out the optimizations for the docking parameters and scoring functions in advance.

The crystal structures of the unphosphorylated S6K1 kinase (PDB: 3A60) domain bound to staurosporine was selected as reference receptor since it has a higher resolution (2.80 Å). The root mean square deviation (RMSD) value between the docked and bound ligand in the crystal structure was used to optimize docking parameters. After many runs, the final optimized parameters could produce a very small RMSD value, such as, the “GA parameters” was designed as 7–8 times speed up, the “Number of dockings” was set to ten, the “Detect Cavity” and “Solvate all” were defined as true, respectively. The “Early termination” was selected as false, the “Flip Planar R-NR1R2” was turn off, and the rest parameters were kept at their default values.

In order to select an appropriate scoring function, a set of known S6K1 inhibitors (inhibitory activity range of three orders) were docked into the active site of S6K1 using our previously optimized docking parameters. The correlation coefficient between the experimentally measured IC50 values and the four scoring functions (GoldScore, ChemScore, ASP and ChemPLP) values were calculated, respectively. We found that GoldScore gave the best correlation coefficient. Therefore, GoldScore was gave used in subsequent DB-VS studies.

### Combination of PB-VS, GB-VS, and DB-VS for database screening

**Fig. 4:**
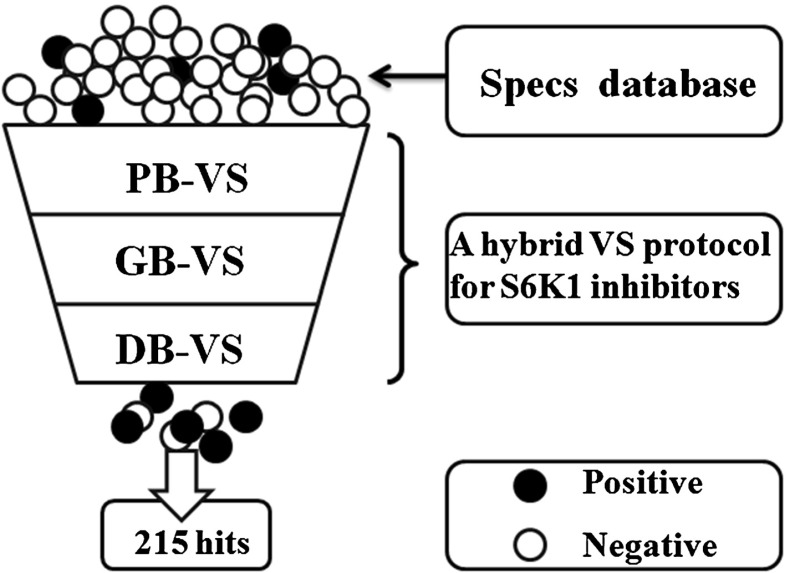
A hybrid VS protocol based on pharmacophore hypothesis, genetic function approximation model, and molecular docking was applied to identify novel S6K1 inhibitors and 215 compounds with new scaffolds were selected

The three VS models of S6K1 inhibitors have been successfully constructed. Finally, the three methods have been combined in a hybrid protocol to virtual screen S6K1 inhibitors from the Specs database (202, 408 compounds) (Fig. 4). As shown in Fig. 4, the faster screening method, PB-VS, was used first. Building the 3D pharmacophore model is difficult because these reported S6K1 inhibitors are limited in structural diversity. In order to discover S6K1 inhibitors faster and more accurately, the GFA regression model that deduces the correlation between the selected five descriptors and the biological of present inhibitors was applied to re-filter the PB-VS screened compounds.

Obviously, the PB-VS and GB-VS techniques for S6K1 inhibitors prediction mainly based on the structural information of compounds. Furthermore, the interactions between ligand and active binding site of S6K1 are also considered in the VS process. Thus, the DB-VS method was further applied to re-filter the remaining 5,400 compounds. The following criteria were used in the design of selective kinase inhibitors: (1) compounds have good interactions with the key residues in the active site of S6K1, such as Leu-175, Glu-179, and Met-225, (2) these compounds should have novel scaffolds different from that of the known S6K1 inhibitors, (3) these compounds can be easily purchased from the market. Finally, 215 compounds with promising S6K1 inhibitory activity were selected from the top hits.

**Fig. 5:**
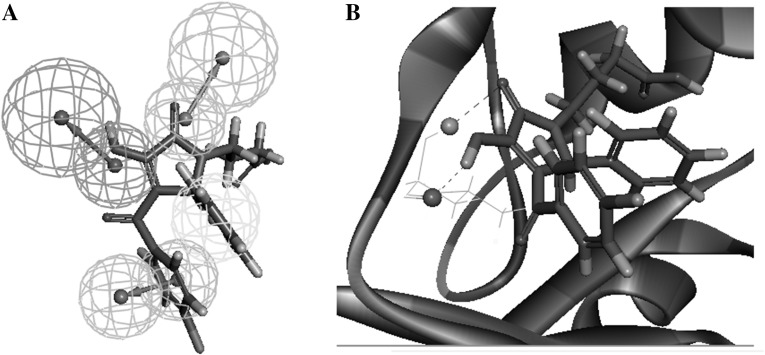
**(A)** One of the final hit compounds, AG227/42189090, mapped with the pharmacophore model Hypo1. **(B)** The binding mode of AG227/42189090 with S6K1 (dashed lines represent hydrogen bonds)

In order to discover S6K1 inhibitors with new scaffolds, we further clustered the 215 compounds into ten classes, and selected 2–7 compounds from each class. Finally, 60 potential active compounds were carefully selected (see Table S1) and have been handed over to other research group to complete the follow-up compound synthesis (or purchase) and activity test, whose results will be reported in the near future. For these promising inhibitors selected by the combined screening method, the representative structures of which mainly include hydrophobic moiety, ring-aromatic moiety, hydrogen bond donor feature and hydrogen bond acceptor feature. Furthermore, the hydrophobic moiety and ring-aromatic moiety of inhibitors situate at hydrophobic pocket of the S6K1 protein. The hydrogen bond donor feature and hydrogen bond acceptor feature of inhibitors form hydrogen-bonds to the sidechains of S6K1. Moreover, all the selected inhibitors are predicted to have good interactions with the active site of residues of Leu-175, Glu-179, and Met-225. For example, Fig. 5 shows the pharmacophore mapping and binding mode of one of the best retrieved compounds. As can be seen from the Fig. 5A, the hit compound AG227/42189090 (4-[3-(4-chlorobenzoyl)-2-(2-fluorophenyl)-4-hydroxy-5-oxo-2,5-dihydro-1H-pyrrol-1-yl]butanoic acid) is mapped very well with these features of the Hypo1. The binding pose of AG227/42189090 in the ATP binding pocket is also shown in Fig. 5B. The 2-fluorophenyl moiety (hydrophobic feature) and 4-chlorobenzoyl group (ring-aromatic feature) make many favorable van der Waals contacts with the backbone and side chains of residues. The hydrogen bond acceptor feature in Hypo1 corresponds to the hydrogen bond interaction formed between the carboxyl pyrrol of the AG227/42189090 and amide nitrogen of Leu-175 in the linker region. The hydrogen bond donor feature in Hypo1 corresponds to the hydrogen bond interaction formed between the 4-hydroxy of AG227/42189090 and carboxyl of Leu-175. The butanoic acid group is directed toward the solvent accessible region. The observed van der Waals interactions and characteristic polar contacts are consistent with the Ye et al [17] provided a possible pharmacophore model of S6K1 inhibitors.

## Conclusions

In this study, a hybrid VS method, including pharmacophore hypothesis, GFA model and molecular docking, has been developed and applied to identify S6K1 inhibitors with new scaffolds. We first developed a common feature pharmacophore hypotheses and GFA regression model of S6K1 inhibitors. These two models were then used to screen the Specs database for the identification of potential new S6K1 inhibitors. Then, a molecular docking method was used to further filter these screened compounds. Finally, 60 potential active compounds were carefully selected from the final hits and have been assigned to another research group to complete the follow-up compound synthesis (or purchase) and activity test.

## Electronic supplementary material

Below is the link to the electronic supplementary material.

Supplementary material 1 (doc 74 KB)
