# Supplementary material for: Combination of pharmacophore hypothesis, genetic function approximation model, and molecular docking to identify novel inhibitors of S6K1
Source: Mol Divers. 2013 Aug 28;17(4):767–72. doi: 10.1007/s11030-013-9473-7 (PMC3824193; doi:10.1007/s11030-013-9473-7)
Supplement: Supplementary file 1 — Supplementary material 1 (doc 74 KB) [file 11030_2013_9473_MOESM1_ESM.doc]

**Table S1.** Chemical structures of the top 60 compounds obtained in the virtual screening using the multistep VS method, including pharmacophore hypothesis, GFA model and molecular docking.

| NO | Name | Chemical structure (in SMILES format) |
| --- | --- | --- |
| 1 | T5841049 | CC(=O)N[C@@H]1[C@H](O)[C@@H](O)[C@@H](CO)O[C@H]1Oc2ccccc2CC=C |
| 2 | T5292835 | C[C@H](NC[C@@H](O)COc1cc(Cl)ccc1Cl)c2cccc(Cl)c2 |
| 3 | T6325987 | CCOc1ccc(OCCNC(=O)[C@@H](Cc2ccccc2)NC(=O)N)cc1 |
| 4 | T5306919 | Oc1cc(Cl)ccc1C(=O)OCC(=O)c2ccc(F)cc2 |
| 5 | T0506-1016 | C[C@@H](NC[C@@H](O)COc1cc(C)c(Cl)c(C)c1)c2ccccc2 |
| 6 | T6509948 | COc1ccc(OC)c(c1)[C@@H](C)NC(=O)CO\N=C\c2ccc(OC)c(OC)c2 |
| 7 | T6590273 | O[C@@H](CNC(=O)CCCc1nc2ccccc2[nH]1)c3cccc(Cl)c3 |
| 8 | T6426323 | CSc1nc(C)c(CCC(=O)Nc2cc(nn2C)C(C)(C)C)c(C)n1 |
| 9 | T6330424 | Cc1cc(C)c(C#N)c(SCC(=O)NCc2ccc(cc2)S(=O)(=O)N)n1 |
| 10 | AN-465#42888489 | COc1cc(CNC[C@@H](O)c2ccccc2)ccc1OCC(=O)N |
| 11 | T5822167 | CN1C(=O)Cc2cc(ccc12)S(=O)(=O)NCc3ccc(Cl)cc3Cl |
| 12 | AG-690#12136162 | COC(=O)CCC(=O)Nc1ccc(cc1)S(=O)(=O)Nc2cc(C)on2 |
| 13 | AN-465#41674938 | COC(=O)c1ccccc1NC(=O)CSc2nnc(NC(=O)C)s2 |
| 14 | T6078699 | NC(=O)[C@@H](OC(=O)COc1ccc2CCCc2c1)c3ccccc3 |
| 15 | T5803832 | COC(=O)[C@@H](Cc1ccc(O)cc1)NC(=O)CCNC(=O)c2ccccc2 |
| 16 | AG-227#42189090 | OC(=O)CCCN1[C@H](C(=C(O)C1=O)C(=O)c2ccc(Cl)cc2)c3ccccc3F |
| 17 | AN-648#37428007 | Nc1nonc1c2nc3ccccc3n2Cc4ccccc4C#N |
| 18 | T6011426 | COC(=O)c1cccnc1NCCc2ccc(cc2)S(=O)(=O)N |
| 19 | AN-329#13029301 | C[C@@H](Oc1ccc(Cl)cc1Cl)C(=O)Nc2ccc(cc2)S(=O)(=O)Nc3nc(C)cc(C)n3 |
| 20 | T0506-9278 | COc1cc(ccc1OCc2ccccc2)[C@H]3Nc4cc(Cl)c(cc4S(=O)(=O)N3)S(=O)(=O)N |
| 21 | T5647963 | CCCOc1ccc(NC=C2C(=O)NC(=S)NC2=O)cc1OCCC |
| 22 | T6487667 | NS(=O)(=O)c1ccc2NC(=O)\C(=C\c3oc(cc3)c4ccc(Cl)cc4)\c2c1 |
| 23 | T5519728 | O=C(NCCc1c[nH]c2ccccc12)c3ccc(NS(=O)(=O)c4ccc5OCCOc5c4)cc3 |
| 24 | T5770199 | CCOC(=O)c1sc(NC(=O)c2ccc3[nH]cnc3c2)cc1C |
| 25 | AM-807#13616345 | COc1ccc(cc1OC)c2ccc(C#N)c(SCC(=O)Nc3ncc(C)s3)n2 |
| 26 | T5920157 | O=C(NCc1csc(n1)c2ccccc2)C3=Cc4ccccc4NC3=O |
| 27 | T6463164 | COC(=O)c1cnc(NC(=O)Cc2csc(Cc3ccccc3)n2)s1 |
| 28 | AN-329#41876874 | Cc1onc(NS(=O)(=O)c2ccc(NC(=O)COc3ccc(Cl)c(C)c3)cc2)c1 |
| 29 | AR-422#41674182 | Nc1ncnc2[nH]c(SCc3ccc(Cl)cc3Cl)nc12 |
| 30 | AR-422#41227859 | CCOc1ccc(OCCn2c(SCC(=O)O)nc3c(N)ncnc23)cc1 |
| 31 | T6275722 | NC(=O)c1ccc(COC(=O)c2cc(n[nH]2)c3ccc(Cl)cc3)cc1 |
| 32 | AP-853#42938734 | O=C(CSc1oc(c2ccccc2)c(n1)c3ccccc3)Nc4nncs4 |
| 33 | AH-487#40686374 | Cc1ccc(cc1)S(=O)(=O)N\N=C\c2ccc(OCc3ccccc3F)cc2 |
| 34 | T5920464 | FC(F)(F)COc1ccc(cn1)C(=O)NCCCc2nc3ccccc3[nH]2 |
| 35 | AG-690#33365034 | C(CCCc1nc2ccccc2[nH]1)CCCc3nc4ccccc4[nH]3 |
| 36 | AN-648#15098015 | COc1ccc2[nH]c(SCc3cccc(I)c3)nc2c1 |
| 37 | T5750610 | CCCn1c(NC(=O)c2ccc(CNC(=O)C)cc2)nc3ccccc13 |
| 38 | T5823136 | COc1cccc2sc(NC(=O)c3ccc(s3)[N+](=O)[O-])nc12 |
| 39 | T6469615 | CC(C)c1ccc(SCC(=O)c2ccc(NS(=O)(=O)C)cc2)cc1 |
| 40 | T6121175 | NS(=O)(=O)c1ccc(NC(=O)CCc2c([nH]c3ccccc23)c4ccc(F)cc4)cc1 |
| 41 | T6422877 | Cc1cc(C)nc(Sc2ccc(NC(=O)CN3C=CC(=O)NC3=O)cc2)n1 |
| 42 | T6109380 | O=C(NNC(=O)c1csc(n1)c2ccccc2)c3cccnc3 |
| 43 | AQ-405#42300434 | CCCCc1nc2ccccc2n1Cc3ccc(cc3)c4ccccc4c5nn[nH]n5 |
| 44 | T0507-7095 | Cc1ccsc1C(=O)Nc2nnc(SCc3ccc(Cl)cc3)s2 |
| 45 | T6285954 | Cc1ccc(SCCNC(=O)c2ccc(N)c(c2)[N+](=O)[O-])cc1 |
| 46 | T5686210 | COc1ccc(CCNC(=O)c2cccc(c2)[N+](=O)[O-])cc1 |
| 47 | AI-204#31685060 | Nc1nnc(SCCCN2C(=O)Nc3ccccc23)s1 |
| 48 | T6084052 | [O-][N+](=O)c1ccc(NCCSc2ncc(cc2Cl)C(F)(F)F)nc1 |
| 49 | T6179698 | O=C(Cc1ccc(cc1)c2ccccc2)Nc3ccc4NC(=O)C(=O)Nc4c3 |
| 50 | AN-988#41872897 | CCc1nnc(NC(=O)CCN2C(=O)c3ccccc3S2(=O)=O)s1 |
| 51 | AG-205#36591025 | COc1cccc(Nc2nc(cs2)c3cccc(c3)N4C(=O)c5ccccc5C4=O)c1 |
| 52 | T6103893 | O=C(Cc1ccc(cc1)c2ccccc2)Nc3ccc4NC(=O)Nc4c3 |
| 53 | AO-476#40672092 | Clc1cccc(NC(=O)Nc2n[nH]c3nnc(c4ccccc4)c(c5ccccc5)c23)c1 |
| 54 | AR-434#42807972 | Cc1sc2N=C(CS(=O)(=O)c3ccccc3)NC(=O)c2c1c4ccccc4 |
| 55 | AF-399#42217126 | Cc1ccc2oc(C(=O)NCc3ccc(cc3)S(=O)(=O)N)c(C)c2c1 |
| 56 | T6060658 | FC(F)(F)Oc1ccc2nc(NC(=O)CCc3occc3)sc2c1 |
| 57 | T5221343 | O=C(Nc1nnc(s1)C2CC2)c3cccc(Oc4ccccc4)c3 |
| 58 | AR-422#41674174 | Nc1ncnc2c1nc(SCc3ccccc3)n2Cc4ccccc4 |
| 59 | AN-329#11481816 | Oc1cc2ccccc2cc1C(=O)N\N=C\c3cc(ccc3Cl)[N+](=O)[O-] |
| 60 | AG-690#11632037 | CCc1nnc(NS(=O)(=O)c2ccc(N\C=C(/C=O)\c3oc4ccccc4n3)cc2)s1 |
